# α-d-2′-De­oxy­adenosine, an irradiation product of canonical DNA and a com­ponent of anomeric nucleic acids: crystal structure, packing and Hirshfeld surface analysis

**DOI:** 10.1107/S2053229624000457

**Published:** 2024-01-22

**Authors:** Peter Leonard, Aigui Zhang, Simone Budow-Busse, Constantin Daniliuc, Frank Seela

**Affiliations:** a Laboratory of Bioorganic Chemistry and Chemical Biology, Center for Nanotechnology, Heisenbergstrasse 11, 48149 Münster, Germany; b Organisch-Chemisches Institut, Westfälische Wilhelms-Universität Münster, Corrensstrasse 40, 48149 Münster, Germany; c Laboratorium für Organische und Bioorganische Chemie, Institut für Chemie, Universität Osnabrück, Barbarastrasse 7, Osnabrück 49069, Germany; University of Notre Dame, USA

**Keywords:** α-2′-de­oxy­adenosine, anomer, crystal structure, crystal packing, nucleoside, Hirshfeld surface analysis

## Abstract

α-d-2′-De­oxy­ribonucleosides are products of the γ-irradiation of DNA under oxygen-free conditions and are constituents of anomeric DNA. They are not found as natural building blocks of canonical DNA. Reports on their conformational properties are limited. Herein, the single-crystal X-ray structure of α-d-2′-de­oxy­adenosine (α-dA), C_10_H_13_N_5_O_3_, and its conformational parameters were determined. In the crystalline state, α-dA forms two conformers in the asymmetric unit which are connected by hydro­gen bonds. The sugar moiety of each conformer is arranged in a ‘clamp’-like fashion with respect to the other conformer, forming hydro­gen bonds to its nucleobase and sugar residue. For both conformers, a *syn* conformation of the nucleobase with respect to the sugar moiety was found. This is contrary to the *anti* conformation usually preferred by α-nucleosides. The sugar conformation of both conformers is C2′-*endo*, and the 5′-hydroxyl groups are in a +*sc* orientation, probably due to the hydro­gen bonds formed by the conformers. The formation of the supra­molecular assembly of α-dA is controlled by hydro­gen bonding and stacking inter­actions, which was verified by a Hirshfeld and curvedness surface analysis. Chains of hydro­gen-bonded nucleobases extend parallel to the *b* direction and are linked to equivalent chains by hydro­gen bonds involving the sugar moieties to form a sheet. A com­parison of the solid-state structures of the anomeric 2′-de­oxy­adenosines revealed significant differences of their conformational parameters.

## Abstract 2

α-d-2′-De­oxy­adenosine, the anomer of the canonical nucleoside β-d-2′-de­oxy­adenosine, is a γ-irradiation product of DNA and a constituent of anomeric DNA. The single-crystal X-ray analysis reveals the formation of two conformers with unusual conformational parameters for α-nucleosides. Hydrogen-bonding and stacking inter­actions form a tightly packed supra­molecular assembly that was verified by Hirshfeld surface analysis.

## Introduction

**Figure 1:**
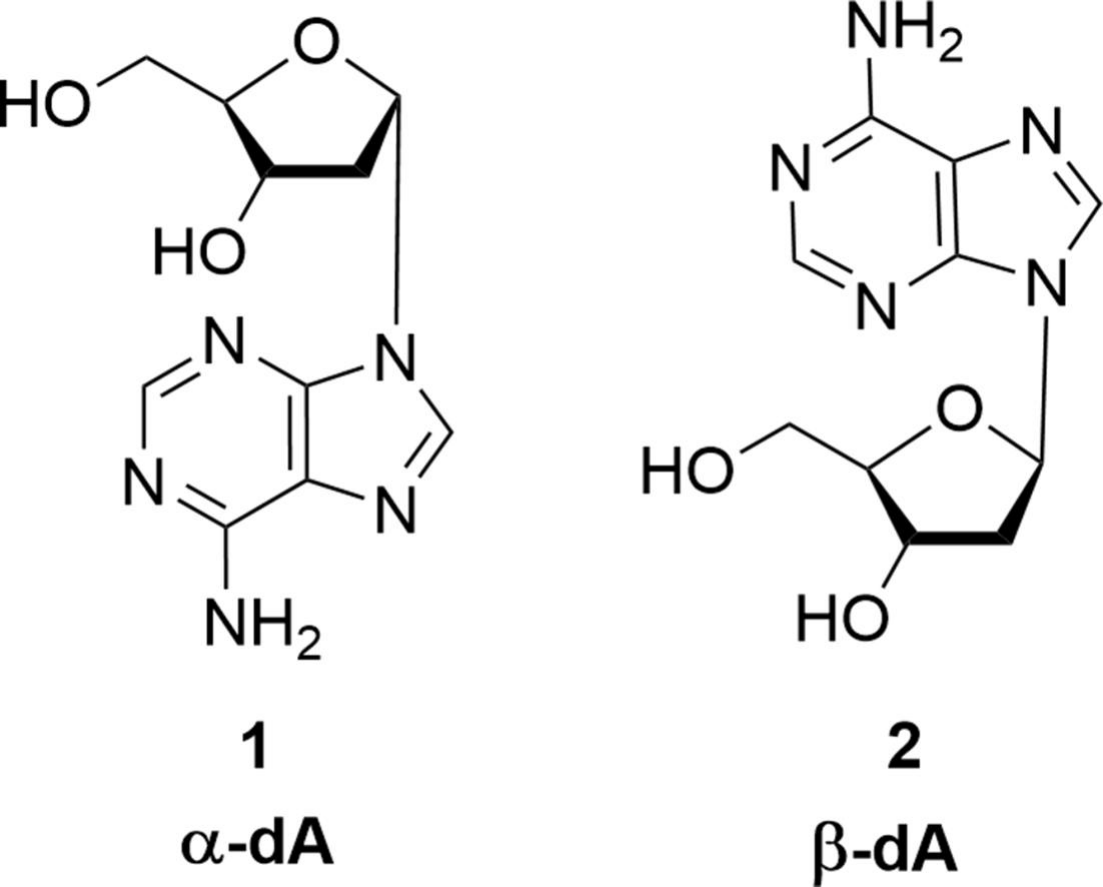
Structures of anomeric 2′-de­oxy­adenosines.

In canonical double-stranded DNA, all four canonical nucleosides display a β-d configuration; α-anomeric nucleosides are not found in native DNA. However, γ-irradiation of DNA under oxygen-free (anoxic) conditions can cause lesions that yield α-d nucleosides as single or multiple mutations (Amato & Wang, 2014 ▸). This is explained by the formation of C1′ radicals at the 2′-de­oxy­ribose moiety formed during irradiation and the nonstereospecific recombination resulting in a mixture of anomers (β and α). Lesiak & Wheeler (1990 ▸) reported the formation of α-2′-de­oxy­adenosine (α-dA) (Fig. 1 ▸) as a major lesion product upon γ-irradiation of poly-dA–poly-dT duplexes and salmon testis DNA under a nitro­gen atmosphere. These α-dA mutations can have a significant impact on DNA stability, which strongly depends on the nearest neighbours (Ide *et al.*, 1995 ▸; Johnson *et al.*, 2012 ▸). The NMR solution structure of a DNA duplex con­taining an α-dA modification revealed local helical changes at the modification site depending on the sequence context (Aramini *et al.*, 2004 ▸). However, com­pared to other types of damage, this type of DNA damage perturbs the DNA helix only to a minor extent and, as a consequence, recognition by repair enzymes is very challenging (Johnson *et al.*, 2012 ▸). The enzymatic repair machinery for α-anomeric lesions is conserved in mammalian cells which shows the biological significance (Johnson *et al.*, 2012 ▸).

Our laboratory and others constructed oligonucleotides con­taining exclusively α-nucleosides (Morvan *et al.*, 1987*a* ▸,*b* ▸; Paoletti *et al.*, 1989 ▸; Chai *et al.*, 2020 ▸; Zhang *et al.*, 2022*a* ▸). They were hybridized with com­plementary strands with all the nucleoside residues in a β-d conformation. These duplexes are as stable as those of canonical DNA with both strands in a β-d configuration. However, strands are in a parallel and not an anti­parallel orientation as in natural DNA. Moreover, α-oligonucleotides were used as invader strands in DNA displacement reactions (Zhang *et al.*, 2022*b* ▸) or implemented in the construction of α/β-heterochiral DNA duplexes con­taining silver-mediated base pairs (Chai *et al.*, 2020 ▸).

The chemical synthesis of nucleosides often produces α-d anomers as by-products during glycosyl­ation. The outcome of the glycosyl­ation reaction (α/β ratio) can be influenced by the structures of the starting materials and the experimental conditions. Various methods were established to synthesize α-d nucleosides by anomerization of β-d anomers or by stereoselective synthesis (Ni *et al.*, 2019 ▸). Thus, a number of α-d nucleosides were prepared, including those with a modified nucleobase or sugar moiety. Due to the difference in the configuration at C1′, monomeric α-d nucleosides show altered properties com­pared to their β-anomers (Ciuffreda *et al.*, 2007 ▸). According to the work of Sundaralingam (1971 ▸) and Latha & Yathindra (1992 ▸) on the solid-state conformations of α-nucleosides, the flexibility of the glycosylic bond, connecting the nucleobase and the sugar moiety, as well as the sugar conformation, seems to be more restricted com­pared to β-nucleosides. However, examples of α-nucleosides with properties outside the proposed favoured conformational range were reported recently (Seela *et al.*, 2002 ▸; Budow-Busse *et al.*, 2021 ▸).

A number of X-ray studies were performed to elucidate the solid-state structure of α-nucleosides, but to our surprise, from the four α-d-2′-de­oxy­ribonucleosides with canonical nucleobases, only X-ray studies of the pyrimidine nucleosides α-2′-de­oxy­thymidine (α-dT) (Görbitz *et al.*, 2005 ▸) and α-2′-de­oxy­cytidine (α-dC) (Budow-Busse, *et al.*, 2021 ▸) have been re­ported. The crystal structure of the anomeric purine nucleoside α-2′-de­oxy­adenosine (α-dA, **1**) (Fig. 1 ▸) is still unknown. Only a preliminary X-ray analysis of a com­plex of human endonucleoase 1 (APE1) with an oligonucleotide con­taining α-dA, **1**, was published by Retailleau *et al.* (2010 ▸).

To obtain more knowledge of crystal structures of α-d-2′-de­oxy­ribonucleosides with a canonical nucleobase, we per­formed a single-crystal X-ray analysis of α-d-2′-de­oxy­ade­nosine (**1**). α-d-2′-De­oxy­adenosine (**1**) had been synthesized previously by Ness and Fletcher in 1960 (Ness & Fletcher, 1960 ▸), and improved synthetic methods were reported by Robins (Robins & Robins, 1965 ▸) and Shinozuka (Shinozuka *et al.*, 1992 ▸).

The work described herein is the first study of the crystal structure of an α-anomeric canonical purine 2′-de­oxy­ribonucleoside. The crystal structure of α-dA (**1**) revealed an unexpected *syn* conformation of the nucleobase. The solid-state conformational properties of **1** were studied in detail and com­pared to those of the corresponding β-d nucleoside (β-dA, **2**) (Fig. 1 ▸) (Sato, 1984 ▸). The inter­actions of the mol­ecules within the crystalline network were analysed. Hirshfeld surface analyses were performed for both anomers (α-dA, **1**, and β-dA, **2**) to support the X-ray data.

## Experimental

### Synthesis and crystallization of α-2′-de­oxy­adenosine (1)

α-2′-De­oxy­adenosine (**1**) was synthesized following the glycosyl­ation protocol reported by Ness (1968 ▸). *N*-Benzoyl­adenine and Hoffer’s chloro­sugar (Hoffer, 1960 ▸) were used as starting materials and, in our hands, a 1:1 anomeric mixture of the protected α-dA and β-dA was obtained. Deprotection in 0.2 *M* NaOMe (room temperature, overnight) afforded α-dA (**1**).

For crystallization, com­pound **1** was dissolved in methanol and was obtained as colourless plates by slow evaporation of the solvent (room temperature, 8 d). A plate-like specimen of **1** was used for the X-ray crystallographic analysis.

### X-ray diffraction and refinement

Crystal data, data collection and structure refinement details are summarized in Table 1 ▸. All carbon-bound H atoms were placed in idealized positions and refined using a riding model, with C—H = 0.95 Å for aromatic CH groups, 0.99 Å for secondary CH_2_ groups and 1.00 Å for tertiary CH groups, using *U*_iso_(H) = 1.2*U*_eq_(C). The positions of the H atoms at N6*A*, N6*B*, O3′*A*, O3′*B*, O5′*A* and O5′*B* were located in a difference map and were refined freely.

## Results and discussion

### Mol­ecular geometry and conformation of α-2′-de­oxy­adenosine (1)

**Figure 2:**
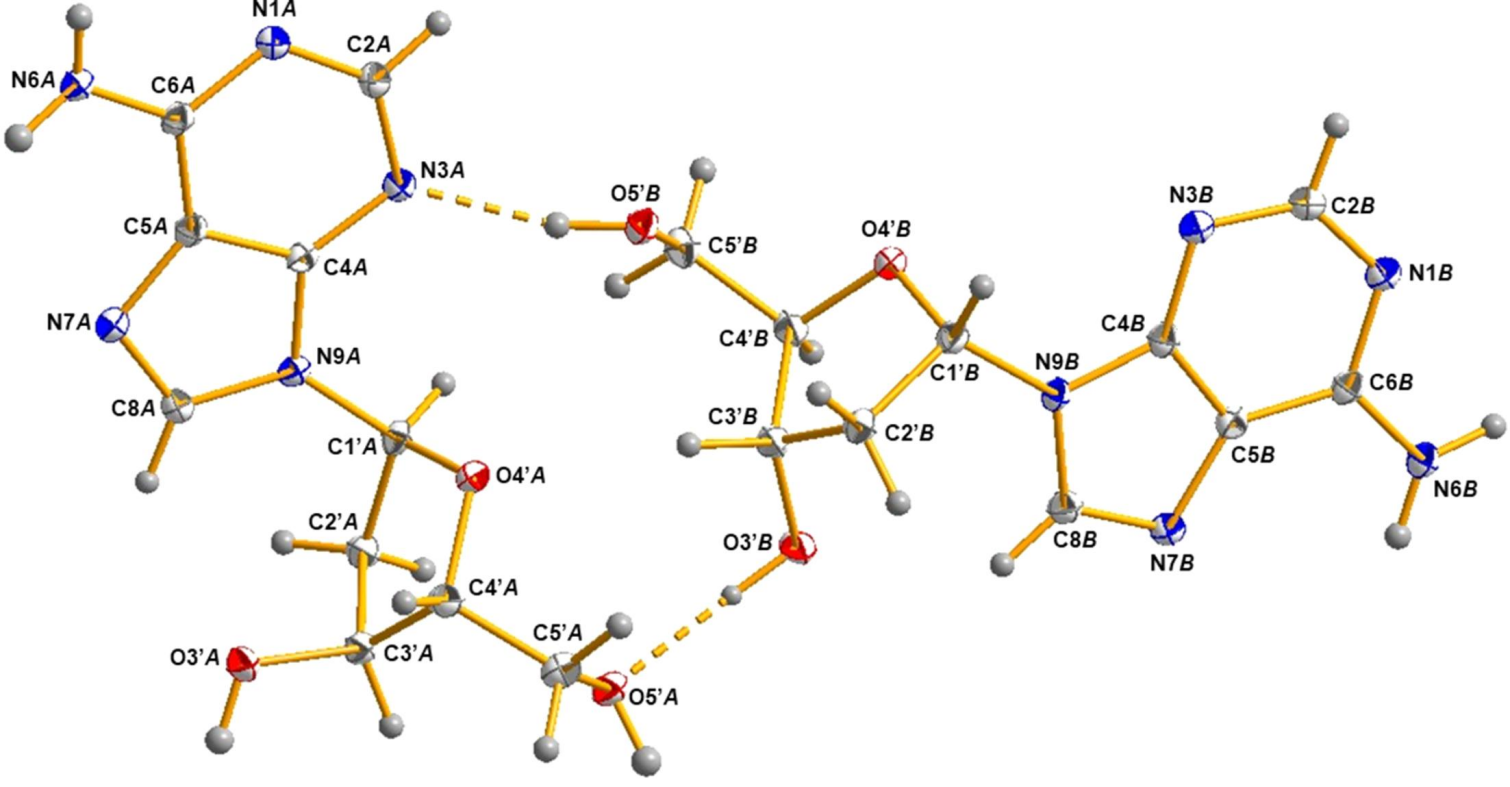
Perspective view and atomic numbering scheme of the two conformers of α-dA (α-**1a** and α-**1b**), being connected to each other by two hydro­gen bonds (dashed lines). Displacement ellipsoids are drawn at the 50% probability level and H atoms are shown as small spheres of arbitrary size.

The crystals of α-2′-de­oxy­adenosine (**1**) are triclinic with the space group *P*1 (Table 1 ▸). There are two mol­ecules in the asymmetric unit, denoted as conformer α-**1a** and conformer α-**1b**, which are connected *via* hydro­gen bonds. The three-dimensional (3D) structures of α-**1a** and α-**1b** are shown in Fig. 2 ▸ and indicate an α-orientation of the nucleobases. The anomeric centre at C1′ shows an *S*-configuration, confirming the α-d anomeric structure of **1** which, in addition, is sup­ported by the Flack parameter. Throughout the article, purine numbering is used instead of systematic numbering for the mol­ecules. Selected geometric parameters are summarized in Table 2 ▸.

The crystal structure of β-2′-de­oxy­adenosine monohydrate was reported in 1965 by Watson and co-workers (Watson *et al.*, 1965 ▸), while the crystal structure of anhydrous β-dA (**2**) was published 20 years later (Sato, 1984 ▸). The geometric parameters of β-dA (**2**) (Table 2 ▸) (Sato, 1984 ▸) were used as a com­parison for the two conformers of α-dA (**1**) (α-**1a** and α-**1b**). For the remodelled 3D structure of β-dA (**2**), see Fig. S1 in the supporting information. Remodelling was carried out using the original structure data (CIF file) of **2** (Sato, 1984 ▸) downloaded from the Cambridge Structural Database (CCDC deposition code 1124124; Groom *et al.*, 2016 ▸).

The shape of nucleosides is characterized by four conformational parameters (Saenger, 1984 ▸): (i) the glycosylic torsion angle, (ii) the puckering of the furan­ose ring, (iii) the degree of deviation from planarity of the furan­ose ring and (iv) the orientation of the 5′-hydroxyl group. The geometric parameters (i)–(iv) of conformers α-**1a** and α-**1b** are discussed in the following.

**Figure 3:**
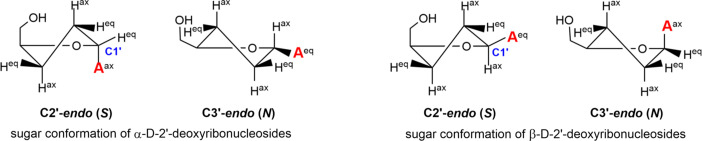
*N* and *S* conformations of α-D and β-D-2′-de­oxy­ribonucleosides in solution. ‘**A**’ corresponds to adenine, ‘ax ’is axial and ‘eq’ is equatorial.

(i) The positioning of the nucleobase with respect to the sugar moiety (*syn*/*anti*) is defined by the torsion angle χ (O4′—C1′—N9—C4) (IUPAC–IUB Joint Commission on Biochemical Nomenclature, 1983 ▸). In the *anti* conformation (180 to ±90°) of purine nucleosides, the six-membered pyrimidine ring is pointing away from the sugar moiety, and there is no particular hindrance of the nucleobase and the sugar residue. On the contrary, in the *syn* conformation (0 to ±90°), the pyrimidine ring is located above the sugar ring, giving rise to close inter­atomic contacts. A C2′-*endo* conformation of the sugar ring reduces the intra­molecular strain, wherein the nucleobase and the C5′ atom are in an equatorial orientation and at a maximum distance (Saenger, 1984 ▸). Purine β-nucleosides are rather flexible and adopt *anti*, high-*anti* and even *syn* conformations, whereas α-nucleosides seem to prefer a rather narrow range of *anti* conformations (Sundaralingam, 1971 ▸; Latha & Yathindra, 1992 ▸). Therefore, we were very surprised to find χ torsion angles of 78.0 (3) and 72.7 (3)° for α-**1a** and α-**1b**, respectively, which are in the range of the *syn* conformation. To the best of our knowledge, this is the first report on α-nucleosides with the nucleobase in a *syn* conformation.

(ii) The five-membered 2′-de­oxy­ribo­furanosyl moiety is nonplanar, with one or two atoms twisted out of plane, referred to as sugar puckering and defined by the phase angle of pseudorotation *P* (Altona & Sundaralingam, 1972 ▸). In general, nucleosides prefer either of the two principal sugar puckering modes, named C3′-*endo* (*N*) or C2′-*endo* (*S*) (Fig. 3 ▸). They correspond to the major displacement of C3′ or C2′ from the median C1′/O4′/C4′ plane and place the more electronegative substituents of C2′ and C3′ in a preferred axial orientation (Saenger, 1984 ▸). β-2′-De­oxy­ribonucleosides favour C2′-*endo* conformations, while α-nucleosides show a preference for C2′-*exo*, C3′-*exo* and C4′-*endo* conformations (Sundaralingam, 1971 ▸; Latha & Yathindra, 1992 ▸). However, in the cases of α-**1a** and α-**1b**, phase angles of pseudorotation of *P* = 135.7° for α-**1a** and *P* = 143.4° for α-**1b** were found. They correspond to C2′-*endo* conformations (Table 2 ▸) and lie clearly outside the preferred conformational range. This is in line with studies on α-2′-de­oxy­cytosine (Budow-Busse *et al.*, 2021 ▸), α-5-acetyl-2′-de­oxy­uridine (Hamor *et al.*, 1977 ▸) and α-5-aza-7-de­aza-2′-de­oxy­guanosine (Seela *et al.*, 2002 ▸), also reporting C2′-*endo* conformations.

(iii) The second parameter used to characterize the geometry of the furan­ose ring is the maximum out-of-plane puckering amplitude τ_m_. The puckering amplitude τ_m_ indicates the degree of deviation from planarity of the furan­ose ring and generally shows an average value of 39° for β-nucleosides, ranging from about 35 to 45° (Altona & Sundaralingam, 1972 ▸). The particular environment of the sugar moiety, *e.g.* hydro­gen-bonding and stacking inter­actions, has an effect on the degree of sugar puckering (small or large τ_m_) and, as a result, the puckering amplitude τ_m_ and the phase angle of pseudorotation *P* are independent parameters. For α-nucleosides, the range of τ_m_ is significantly broadened (28–50°), though the average τ_m_ value remains around 39°. The two conformers α-**1a** and α-**1b** support this finding and adopt τ_m_ values of 30.5 and 31.7°, respectively. These values correspond to a flattening of the 2′-de­oxy­ribose moiety, probably a consequence of hydro­gen-bonding and stacking inter­actions (see next section).

(iv) The conformation around the exocyclic C4′—C5′ bond relative to the sugar ring is determined by the torsion angle γ (O5′—C5′—C4′—C3′) (Saenger, 1984 ▸). The distribution of conformers around the C4′—C5′ bond depends on the nature of the nucleobase and the sugar pucker. However, the difference between the conformational preference about the C4′—C5′ bond of β- and α-nucleosides (Sundaralingam, 1971 ▸) seems to be neglectable. For conformers α-**1a** and α-**1b**, γ torsion angles of 50.2 (3) and 46.8 (3)° are found (Table 2 ▸), corresponding to a +synclinal (+*sc*) conformation. In β-nucleosides, a +synclinal conformation around the exocyclic C4′—C5′ bond, in combination with a *syn* orientation of the nucleobase, positions the nucleobase and atom O5′ above the ribosyl moiety (Saenger, 1984 ▸). For these β-nucleosides, inter­actions between atom O5′ and the nucleobase might pull the torsion angles γ in the +*sc* range. On the contrary, due to the α-anomeric orientation of the nucleobase, this combination (+*sc* and *syn*) leads to an arrangement in α-nucleoside **1** in which atom O5′ is located ‘above’ the sugar residue, while the nucleobase is positioned on the other (‘below’) side of the sugar moiety. As can be clearly seen from Fig. 2 ▸, possible inter­actions between the 5′-hydroxyl group and the nucleobase as in β-nucleosides can be ruled out. Instead, the hydro­gen bonds formed between conformers α-**1a** and α-**1b** might account for the +*sc* orientation of the 5′-hydroxyl groups.

Taken together, both conformers (α-**1a** and α-**1b**) show a high correlation in their overall structural shape (see geometric parameters given in Table 2 ▸). Moreover, it is surprising that although the α-anomeric conformers α-**1a** and α-**1b**, and the canonical β-dA (**2**) differ only in their configuration (α *versus* β), significant differences of the solid-state structures are apparent from their conformational parameters (Table 2 ▸) (for a perspective view of **2**, see Fig. S1 in the supporting information). This is particularly true for the torsion angle χ, the pseudorotational phase angle *P*, the maximum amplitude τ_m_ and the torsion angle γ.

### Crystal packing and hydro­gen bonding

**Figure 4:**
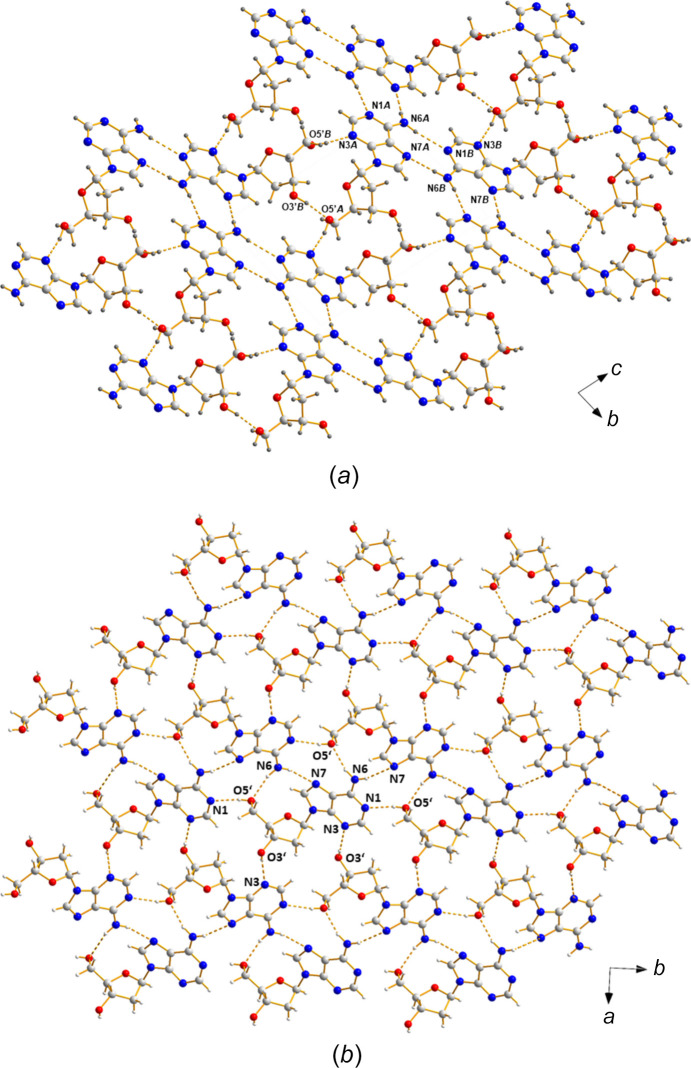
Detailed view of the hydro­gen-bonding schemes (dashed lines) of (*a*) α-dA (**1**) (viewed in the *bc* plane) and (*b*) β-dA (**2**) (viewed in the *ab* plane).

The asymmetric unit of α-2′-de­oxy­adenosine (**1**) con­tains two mol­ecules (α-**1a** and α-**1b**) which show different conformational properties. Both mol­ecules are connected *via* hydro­gen bonds in an unsymmetrical fashion. From the representation of the extended crystalline network shown in Fig. 4 ▸(*a*), it is evident that the sugar moiety of each conformer is arranged in such a way that this moiety functions as a ‘clamp’ with respect to the other conformer, forming hydro­gen bonds to its nucleobase and sugar residue. In detail, the exocyclic 5′-hydroxyl group forms a hydro­gen bond to atom N3 of the nucleobase (O5′*B*—H5′*B*⋯N3*A* and O5′*A*—H5′*A*⋯N3*B* ^v^; for symmetry codes, see Table 3 ▸). The sugar-to-sugar contact is observed between the O3′-hydroxyl group as hydro­gen donor and O5′ as hydro­gen acceptor (O3′*A*—H3′*A*⋯O5′*B* ^v^ and O3′*B*—H3′*B*⋯O5′*A*). In addition, bifurcated hydro­gen bonds are formed by the amino groups, connecting two neighbouring mol­ecules *via* N1 (N6*A*—H01*A*⋯N1*B* ^iii^ and N6*B*—H01*B*⋯N1*A* ^i^) and N7 (N6*A*—H02*B*⋯N7*B* ^iv^ and N6*B*—H02*B*⋯N7*A* ^ii^) of the nucleobase. Despite the fact that conformational differences exist for conformers α-**1a** and α-**1b**, both mol­ecules form hydro­gen bonds with an identical donor–acceptor pattern.

As it was of inter­est to figure out the differences between the anomers of 2′-de­oxy­adenosine in the solid state, images of the crystalline network of β-2′-de­oxy­adenosine (**2**) were generated, using the original CIF file of Sato (1984 ▸). As indicated by Fig. 4 ▸(*b*), the arrangement of the individual mol­ecules of β-dA (**2**) is com­pletely different com­pared to that of the α-anomer (**1**), resulting in another hydro­gen-bonding scheme. In β-dA (**2**), the amino group is in a ‘clamp’-like position with respect to the neighbouring mol­ecule, forming a bifurcated hydro­gen bond to N7 of the nucleobase and O5′ of the sugar moiety as acceptors. Nucleobase-to-sugar contacts use N1 and N3 as hydro­gen acceptors and the exocyclic O3′- and O5′-hydroxyl groups as hydro­gen donors. Contrary to the crystal structure of α-dA (**1**), sugar-to-sugar contacts do not exist in β-dA (**2**).

**Figure 5:**
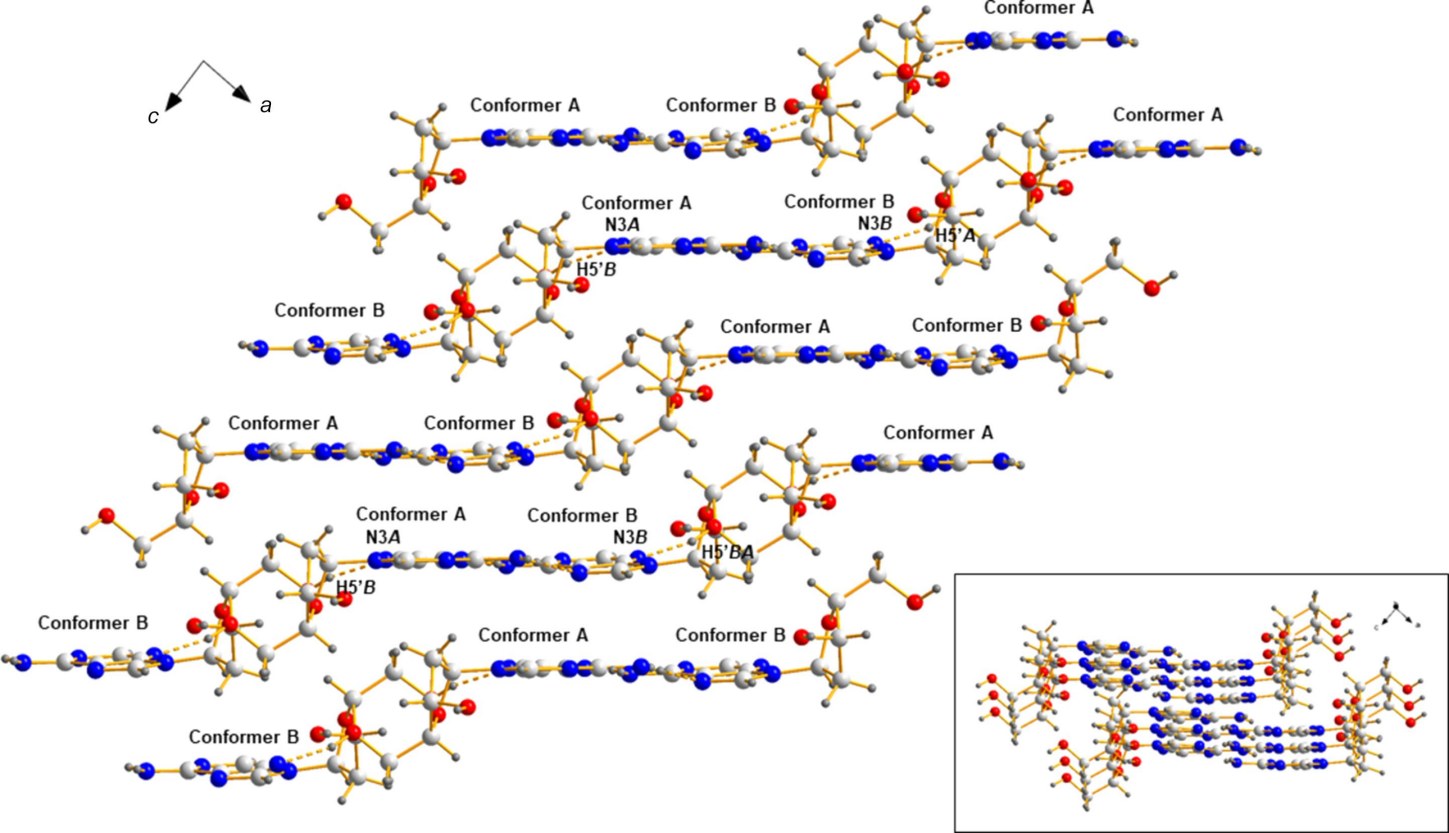
Staircase-like arrangement of conformers α-**1a** and α-**1b** within the extended crystalline network (viewed in the *ca* plane). Inset: stacking inter­actions of the conformers.

Another typical feature of the crystal structures of nucleosides are stacking inter­actions. Due to the aromatic nature of the heterocyclic nucleobase, this moiety is prone to form stacking inter­actions. Fig. 5 ▸ shows that also in the case of α-2′-de­oxy­adenosine (**1**) the nucleobases of the two conformers (α-**1a** and α-**1b**) are stacked. Always one type of conformer forms piles of stacked mol­ecules (see inset of Fig. 5 ▸). Moreover, the nucleobases of α-**1a** and α-**1b** are facing each other, forming a rather flat entity. Accordingly, the sugar moieties of α-**1a** and α-**1b** are also sited opposite each other. The pattern of alternating conformers is continued and the above and below pairs of neighbouring nucleobases are identically positioned (non-alternating), but shifted in such a way that the nucleobases of identical conformers form a staircase-like arrangement. As a result, the two conformers (α-**1a** and α-**1b**) form a highly-ordered packing arrangement.

In the earlier crystal study on β-dA (**2**) of Sato (1984 ▸), no relevant stacking inter­actions were reported. We inspected thoroughly the crystal data of **2** and performed a Hirshfeld surface analysis (for details, see the next section) and found large flat areas in the curvedness plots of **2** which indicate stacking inter­actions. Indeed, remodelling of the extended crystalline network of β-dA (**2**) proves the occurrence of stacking inter­actions of the aromatic nucleobases (see Figs. S3 and S4 in the supporting information).

### Hirshfeld surface analyses of α-2′-de­oxy­adenosine (1) and β-2′-de­oxy­adenosine (2)

**Figure 6:**
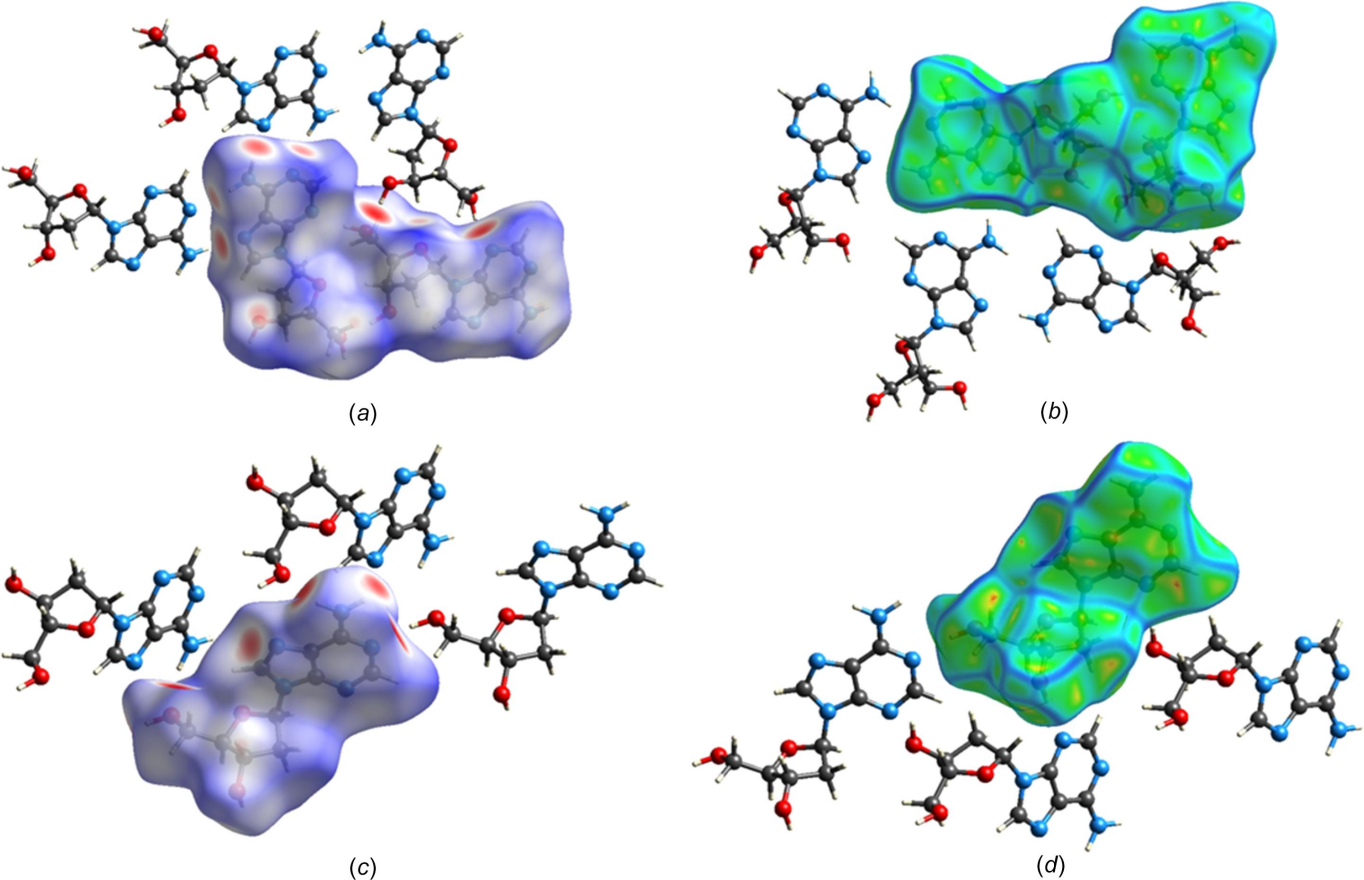
The Hirshfeld surfaces of α-dA (**1**) (upper part) and β-dA (**2**) (lower part) with close-contact mol­ecules, (*a*)/(*c*) mapped over *d*_norm_ (−0.5 to 1.5 Å) and (*b*)/(*d*) mapped over the curvedness (−4.0 to 4.0 Å). Green areas represent flat regions and blue lines indicate edges.

To obtain additional information on the role of crystal packing forces and to visualize the relative strengths of the inter­molecular inter­actions, a Hirshfeld surface analysis of α-2′-de­oxy­adenosine (**1**) was carried out. For this purpose, the program *CrystalExplorer* (Version 17; Spackman & Jayatilaka, 2009 ▸; Turner *et al.*, 2017 ▸) was used. The Hirshfeld surfaces of the two conformers of α-dA (**1**) mapped over a *d*_norm_ range of −0.5 to 1.5 Å are represented, together with close-contact mol­ecules of the central mol­ecules inside the Hirshfeld surface [Fig. 6 ▸(*a*) and Fig. S5 in the supporting information]. Therein, the red surface areas denote strong inter­actions with distances shorter than the sum of the van der Waals radii and negative *d*_norm_, white surface areas represent contacts with distances equal to the sum of the van der Waals radii and blue surfaces refer to weak contacts. Surfaces of the two conformers of **1** plotted over curvedness are shown in Figs. 6 ▸(*b*) and S7 (see supporting information). For shape-index plots, see Fig. S6 in the supporting information.

Inspection of the Hirshfeld surface of conformers α-**1a** and α-**1b** reveals a large unperturbed area [Fig. 6 ▸(*a*)], with the major red spots located on the top view area. These spots correspond to the short-range hydro­gen bonds connecting the sugar moiety of one conformer to the nucleobase and sugar residue of the other conformer, as described in the previous section. Additional and less intense spots indicate the presence of weaker contacts formed by the amino groups. As a result, the Hirshfeld analyses are consistent with the hydro­gen-bonding data (Table 3 ▸). Moreover, the curvedness plot of **1** [Fig. 6 ▸(*b*)] shows large flat areas due to the presence of the aromatic nucleobase and confirms the contribution of stacking inter­actions to the overall crystal packing.

**Figure 7:**
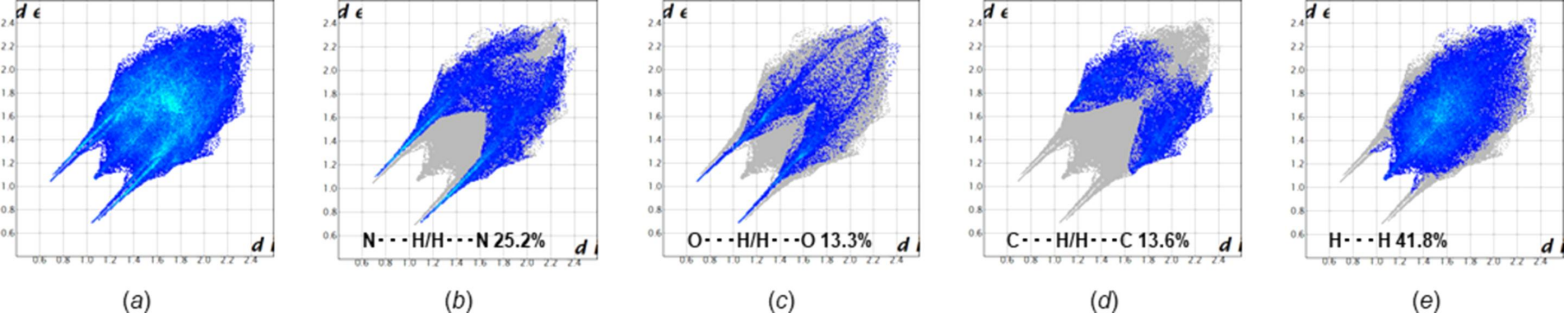
2D fingerprint plots showing the percentage contributions of the various inter­actions to the total Hirshfeld surface area of α-dA (**1**), showing (*a*) all inter­actions and resolved contacts of (*b*) N⋯H/H⋯N, (*c*) O⋯H/H⋯O, (*d*) C⋯H/H⋯C and (*e*) H⋯H.

Two-dimensional (2D) fingerprint plots (Fig. 7 ▸ and Fig. S11 in the supporting information) provide a visual summary of the contribution of each contact type and their relative pro­portion to the Hirshfeld surface. The plots are resolved into N⋯H/H⋯N, O⋯H/H⋯O, C⋯H/H⋯C and H⋯H contacts [Figs. 7 ▸(*b*)–(*e*)]. The N⋯H/H⋯N (25.2%) and O⋯H/H⋯O (13.3%) contacts provide a significant contribution to the surface and form the two characteristic spikes. They represent the strong hydro­gen bonds inter­connecting conformers α-**1a** and α-**1b**. The wings of the plot are occupied by C⋯H/H⋯C inter­actions (13.6%), which include numer­ous weak hydrogen bonds. The nonspecific van der Waals H⋯H contacts (41.8%) occupy the major portion of the surface.

Due to the early publication date of the crystal structure of β-2′-de­oxy­adenosine (**2**) (Sato, 1984 ▸), to the best of our knowledge, a Hirshfeld surface analysis has not yet been performed for β-dA (**2**). Therefore, we also carried out a Hirshfeld surface analysis of **2** [Fig. 6 ▸(*c*) and Fig. S8 in the supporting information] using the crystal structure data (CCDC deposition number 1124124; Sato, 1984 ▸). For shape index and curvedness plots of **2**, see Fig. 6 ▸(*d*) and Figs. S9 and S10 in the supporting information. The Hirshfeld surface analysis of β-dA mapped over *d*_norm_ and curvedness supports the hydro­gen-bonding (Sato, 1984 ▸) and stacking inter­actions of **2**. The calculated 2D fingerprint plots for **2** are shown in Fig. S11 in the supporting information and confirm the significant contribution of the N⋯H/H⋯N (22.2%) and O⋯H/H⋯O (18.7%) contacts to the overall crystal packing forces.

## Conclusion

The present work is the first report on a single-crystal X-ray analysis of an α-d-2′-de­oxy­ribonucleoside carrying a canonical purine nucleobase. In the crystalline state, α-dA forms two conformers (α-**1a** and α-**1b**) in the asymmetric unit which are connected *via* hydro­gen bonds. In contrast to the *anti* conformation commonly preferred by α-nucleosides, the nucleobase moiety of α-**1a** and α-**1b** adopts a *syn* conformation. For both conformers, the sugar conformation is C2′-*endo* and the 5′-hydroxyl group is in a +*sc* orientation.

**Figure 8:**
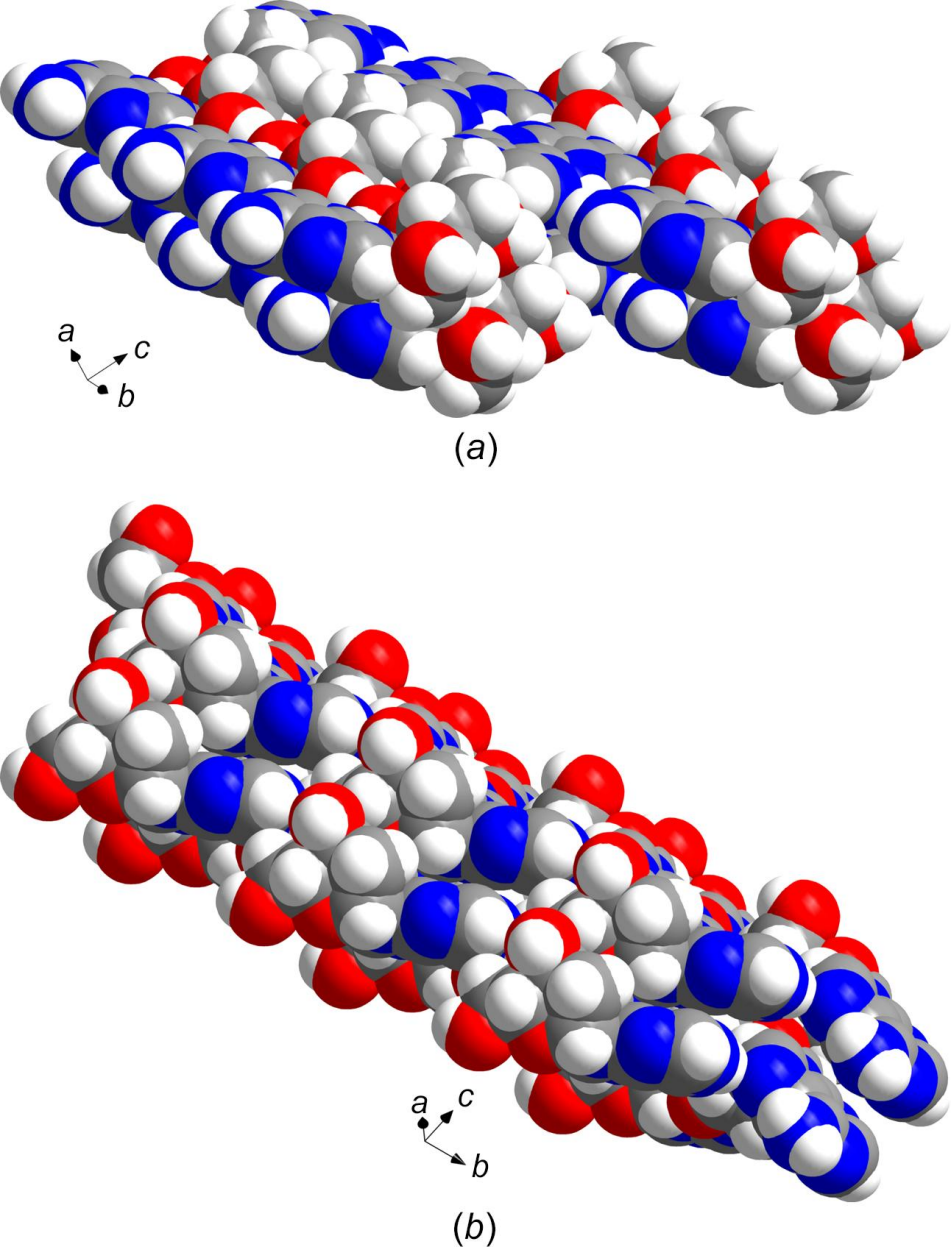
Space-filling models of (*a*) α-dA (**1**) and (*b*) β-dA (**2**).

Comparison of the solid-state structure of α-dA to canonical β-2′-de­oxy­adenosine (**2**) (Sato, 1984 ▸) revealed significant differences in the conformational parameters and in the packing of their supra­molecular networks (see space-filling models in Fig. 8 ▸). In the supra­molecular network of α-dA (**1**), the sugar moieties of each conformer act as clamps by forming hydro­gen bonds to the nucleobases and sugar residues of the other conformer. The nucleobases form hydro­gen-bonded chains which are linked to equivalent chains by hydro­gen bonds involving the sugar moieties to form sheets. The nucleobases and the sugar moieties of alternating α-**1a** and α-**1b** conformers are organized face-to-face with respect to each other. A staircase-like arrangement is formed by the nucleobases of each conformer. In addition, piles of stacked mol­ecules are formed by always one type of conformer. The hydro­gen-bonding pattern is supported by a Hirshfeld surface analysis, and curvedness surfaces confirm the contribution of stacking inter­actions to the overall crystal packing.

α-d-2′-De­oxy­adenosine (**1**) is not found in native DNA, but it is formed as a major lesion product upon γ-irradiation of DNA under anoxic conditions. Furthermore, it is a com­ponent of anomeric DNA formed by one strand in an α-d and the other in a β-d configuration. The latter is as stable as canonical DNA and shows similar base recognition. One of the recent developments in the realm of anomeric DNA is the construction of entirely new nucleic acid structures and the design of new recognition systems to expand the genetic code.

## Supplementary Material

Crystal structure: contains datablock(s) I, global. DOI: 10.1107/S2053229624000457/ov3171sup1.cif

Structure factors: contains datablock(s) I. DOI: 10.1107/S2053229624000457/ov3171Isup2.hkl

Additional packing schemes, Hirshfeld surfaces of the anomers and fingerprint plots. DOI: 10.1107/S2053229624000457/ov3171sup3.pdf

CCDC reference: 2294190

## Figures and Tables

**Table 1:** Experimental details

Crystal data	
Chemical formula	C_10_H_13_N_5_O_3_
*M*_r_	251.25
Crystal system, space group	Triclinic, *P*1
Temperature (K)	100
*a*, *b*, *c* (Å)	5.2027 (2), 8.9738 (3), 12.3715 (4)
α, β, γ (°)	78.249 (1), 83.171 (1), 76.338 (1)
*V* (Å^3^)	547.97 (3)
*Z*	2
Radiation type	Cu *K*α
μ (mm^−1^)	0.98
Crystal size (mm)	0.19 × 0.08 × 0.05

Data collection	
Diffractometer	Bruker D8 Venture Photon III
Absorption correction	Multi-scan (*SADABS*; Krause *et al.*, 2015 ▸)
*T*_min_, *T*_max_	0.84, 0.95
No. of measured, independent and observed [*I* > 2σ(*I*)] reflections	17188, 3858, 3752
*R*_int_	0.041
(sin θ/λ)_max_ (Å^−1^)	0.603

Refinement	
*R*[*F* ^2^ > 2σ(*F* ^2^)], *wR*(*F* ^2^), *S*	0.027, 0.065, 1.08
No. of reflections	3858
No. of parameters	357
No. of restraints	3
H-atom treatment	H atoms treated by a mixture of independent and constrained refinement
Δρ_max_, Δρ_min_ (e Å^−3^)	0.18, −0.17
Absolute structure	Flack *x* determined using 1744 quotients [(*I* ^+^) − (*I* ^−^)]/[(*I* ^+^) + (*I* ^−^)] (Parsons *et al.*, 2013 ▸)
Absolute structure parameter	−0.01 (8)

Computer programs: *APEX4* (Bruker, 2021 ▸), *SAINT* (Bruker, 2019 ▸), *SHELXT2018* (Sheldrick, 2015*a* ▸), *SHELXL2018* (Sheldrick, 2015*b* ▸) and *DIAMOND* (Putz & Brandenburg, 2022 ▸).

**Table 2:** Selected geometric parameters of the anomeric 2′-de­oxy­adenosines α-**1a**, α-**1b** and β-dA (**2**)

	Conformer α-**1a**	Conformer α-**1b**	β-dA (**2**) (Sato, 1984 ▸)
Glycosylic bond length (N9—C1′) (Å)	1.473 (3)	1.474 (3)	1.474 (2)
Torsion angle χ (O4′—C1′—N9—C4) (°)	78.0 (3)	72.7 (3)	−165.0 (7)
Relative sugar/base orientation	*syn*	*syn*	*anti*
Pseudorotational phase angle *P* (°)	135.67	143.35	13.28
Maximum amplitude τ_m_ (°)	30.51	31.68	36.34
Sugar pucker	*S*-type, C2′-*endo*, _1_ *T* ^2^	*S*-type, C2′-*endo*, _1_ *T* ^2^	*N*-type, C3′-*endo*, ^3^ *T*_2_
Torsion angle γ (O5′—C5′—C4′—C3′) (°)	50.2 (3)	46.8 (3)	175.5 (1)
Relative orientation of exocyclic 5′-OH	+synclinal (*gauche*)	+synclinal (*gauche*)	+antiperiplanar (*trans*)

**Table 3:** Hydrogen-bond geometry (Å, °)

*D*—H⋯*A*	*D*—H	H⋯*A*	*D*⋯*A*	*D*—H⋯*A*
N6*B*—H01*B*⋯N1*A* ^i^	0.89 (4)	2.36 (4)	3.239 (3)	170 (1)
N6*B*—H02*B*⋯N7*A* ^ii^	0.86 (4)	2.14 (4)	2.998 (3)	173 (1)
O5′*B*—H5′*B*⋯N3*A*	0.90 (4)	1.91 (4)	2.787 (3)	163 (1)
O3′*B*—H3′*B*⋯O5′*A*	0.90 (4)	1.78 (4)	2.680 (3)	171 (1)
N6*A*—H01*A*⋯N1*B* ^iii^	0.92 (3)	2.33 (3)	3.243 (3)	172 (1)
N6*A*—H02*A*⋯N7*B* ^iv^	0.94 (4)	2.12 (4)	3.056 (3)	178 (1)
O5′*A*—H5′*A*⋯N3*B* ^v^	0.95 (5)	1.85 (5)	2.758 (3)	159 (1)
O3′*A*—H3′*A*⋯O5′*B* ^v^	0.92 (4)	1.80 (4)	2.691 (2)	163 (1)

Symmetry codes: (i) 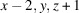 ; (ii) 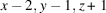 ; (iii) 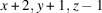 ; (iv) 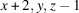 ; (v) 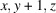 .
