# Supplementary material for: α-d-2′-De­oxy­adenosine, an irradiation product of canonical DNA and a com­ponent of anomeric nucleic acids: crystal structure, packing and Hirshfeld surface analysis
Source: Acta Crystallogr C Struct Chem. 2024 Jan 22;80(Pt 2):21–9. doi: 10.1107/S2053229624000457 (PMC10844955; doi:10.1107/S2053229624000457)
Supplement: Supplementary file 3 [file c-80-00021-sup3.pdf]

STRUCTURAL CHEMISTRY

Volume 80 (2024)

Supporting information for article:

α-D-2′-Deoxyadenosine, an irradiation product of canonical DNA and a component of anomeric nucleic acids: crystal structure, packing and Hirshfeld surface analysis

Peter Leonard, Aigui Zhang, Simone Budow-Busse, Constantin Daniliuc and Frank Seela

Supporting Information

α-D-2’-Deoxyadenosine: an irradiation product of canonical DNA and a component of anomeric nucleic acids. Crystal structure, packing and Hirshfeld surface analysis

Peter Leonard[a], Aigui Zhang[a], Simone Budow-Busse[a], Constantin Daniliuc[b] and Frank Seela[a,c*]

[a]*Laboratory of Bioorganic Chemistry and Chemical Biology, Center for Nanotechnology, Heisenbergstrasse 11, 48149 Münster, Germany*

[b]*Organisch-Chemisches Institut, Westfälische-Wilhelms-Universität Münster, Corrensstrasse 40, 48149 Münster, Germany*

[c]*Laboratorium für Organische und Bioorganische Chemie, Institut für Chemie, Universität Osnabrück, Barbarastrasse 7, 49069 Osnabrück, Germany*

Table of Contents

Figure S1. Perspective view and atomic numbering scheme of β-dA 3

Figure S2. Stacking interactions of the conformers α-**1a** and α-**1b** 3

Figure S3. Extended crystalline network of β-dA 4

Figure S4. Stacking interactions of of β-dA 4

Figure S5. Hirshfeld surface mapped over d_{norm} of α-dA with close contact molecules 5

Figure S6. Hirshfeld surface mapped over the shape index of α-dA 5

Figure S7. Hirshfeld surface mapped over the curvedness of α-dA 6

Figure S8. The Hirshfeld surface mapped over d_{norm} of β-dA with close contact molecules 6

Figure S9. Hirshfeld surface mapped over the shape index of β-dA 7

Figure S10. Hirshfeld surface mapped over the curvedness of β-dA 7

Figure S11. 2D fingerprint plots showing the percentage contributions of the various interactions to the total Hirshfeld surface area of β-dA 8

Reference 8

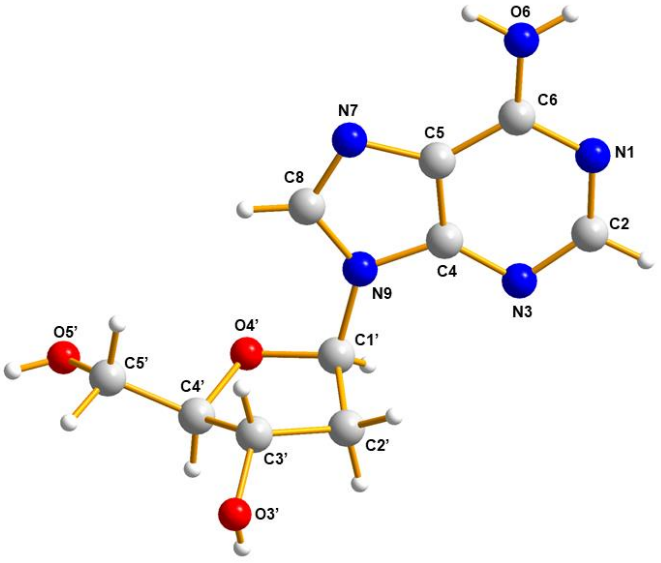

Figure S1. Perspective view and atomic numbering scheme of β-dA (**2**). Displacement ellipsoids are drawn at the 50% probability level and H atoms are shown as small spheres of arbitrary size. The original structure data (cif-file) of **2** (Sato, 1984), downloaded from the Cambridge Structural Database (CCDC 1124124) was used for modelling.

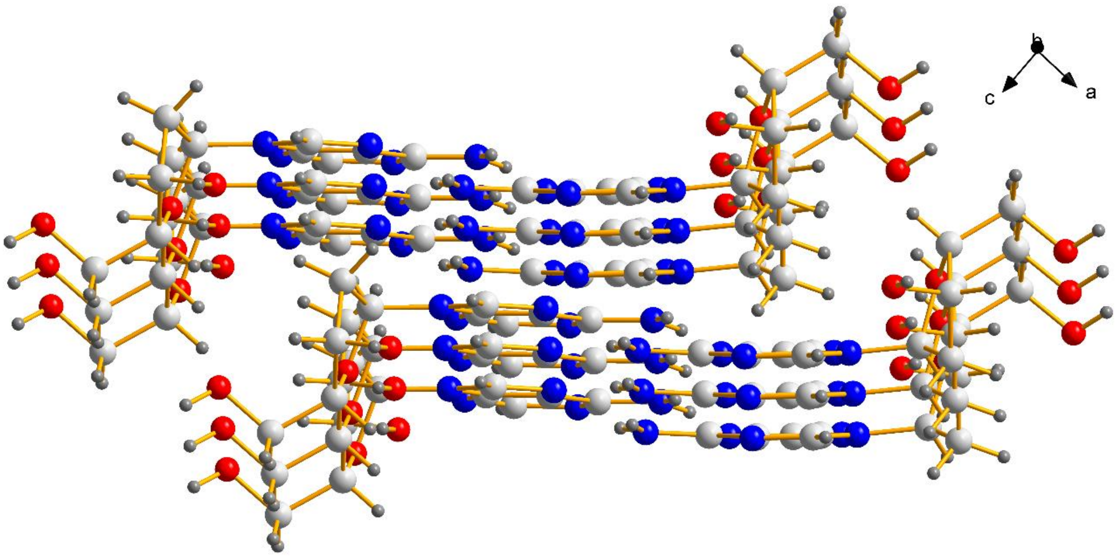

Figure S2. Enlargement of the inset of Figure 5 in the main manuscript: Stacking interactions of the conformers α-**1a** and α-**1b** along the *b*-axis.

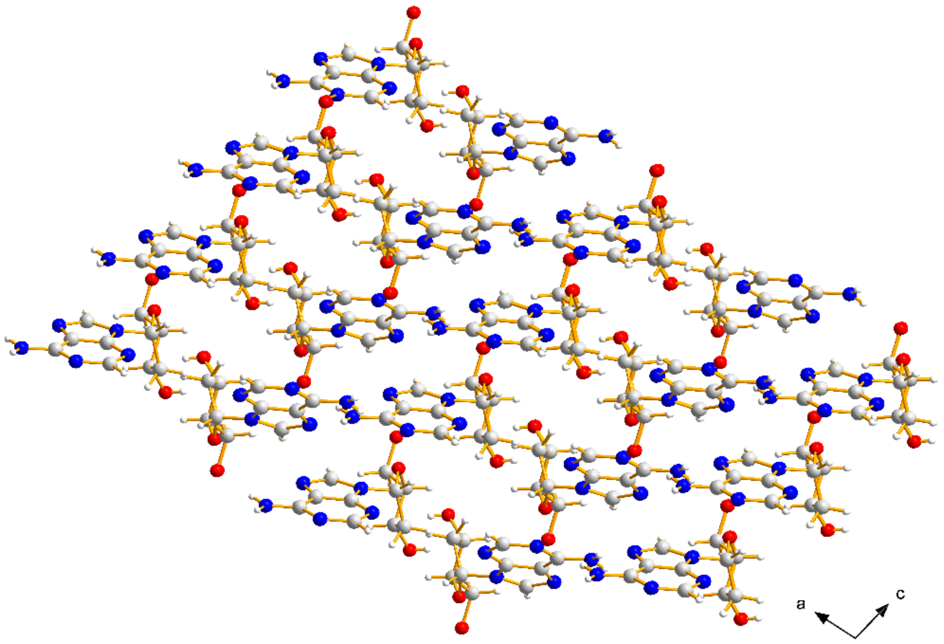

Figure S3. Extended crystalline network of β-dA (**2**) (*ac*-plane).

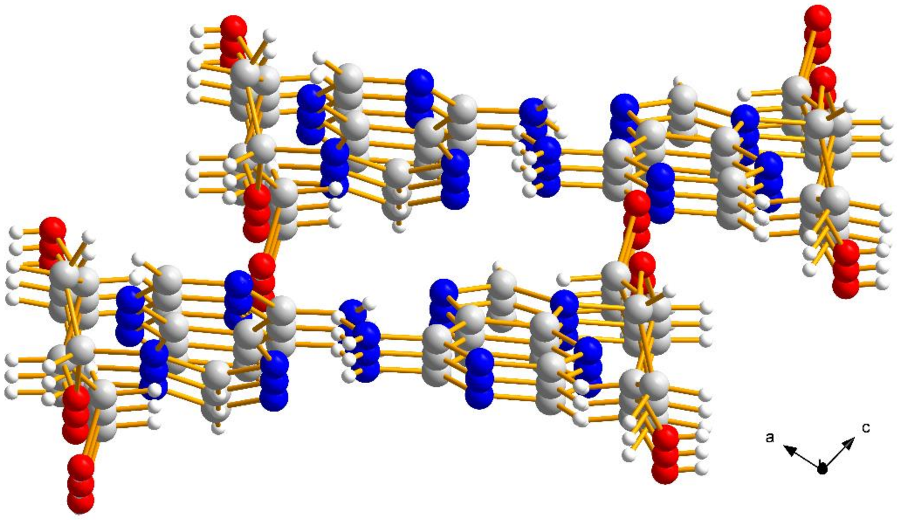

Figure S4. Stacking interactions of of β-dA (**2**) along the *b*-axis.

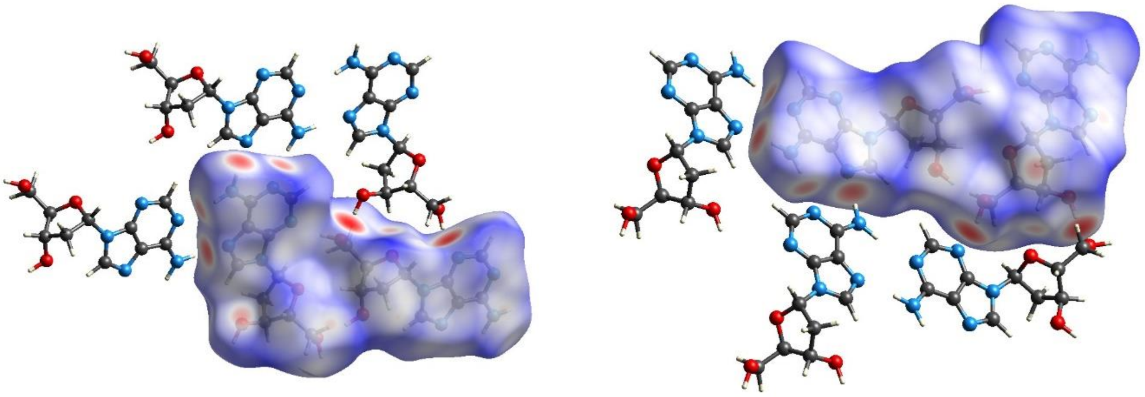

(*a*) (*b*)

Figure S5. Hirshfeld surface of α-dA (**1**) with close contact molecules, mapped over d_{norm} (-0.5 to 1.5 Å). (*a*) Front view. (*b*) Back view.

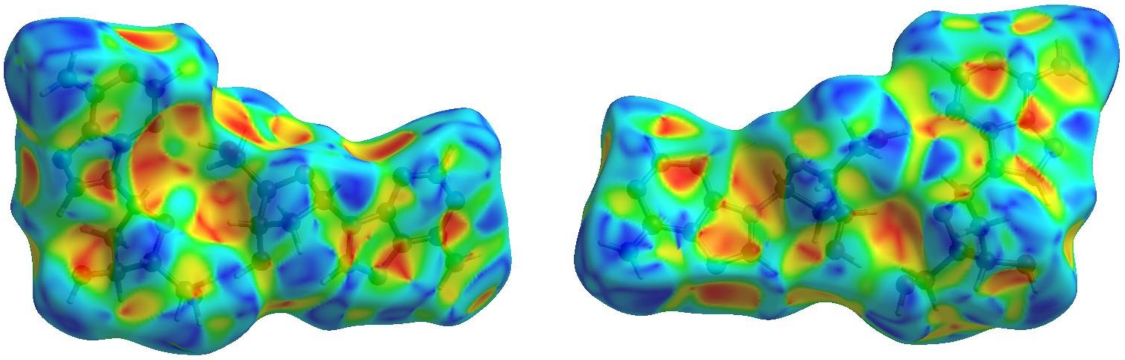

(*a*) (*b*)

Figure S6. Hirshfeld surface mapped over the shape index (-1.0 to 1.0 Å) of the two conformers of α-dA (**1**). (*a*) Front view. (*b*) Back view. Blue areas represent bumps, and red regions indicate hollows.

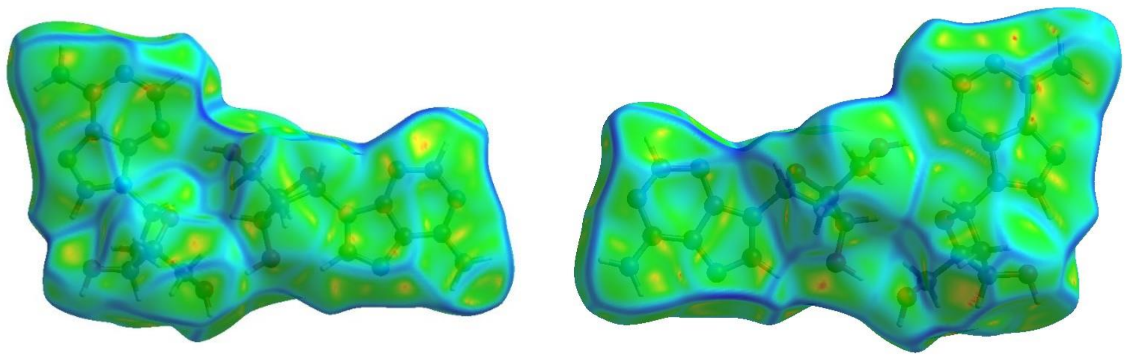

(*a*) (*b*)

Figure S7. Hirshfeld surface mapped over the curvedness (-4.0 to 4.0 Å) of the two conformers of α-dA (**1**). (*a*) Front view. (*b*) Back view. Green areas represent flat regions, and blue lines indicates edges.

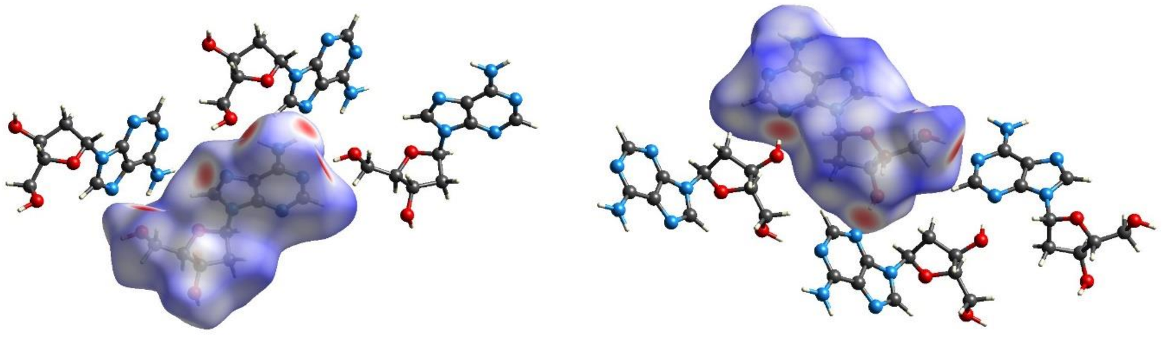

(*a*) (*b*)

Figure S8. The Hirshfeld surface of β-dA (**2**) with close contact molecules, mapped over d_{norm} (-0.5 to 1.5 Å). (*a*) Front view. (*b*) Back view.

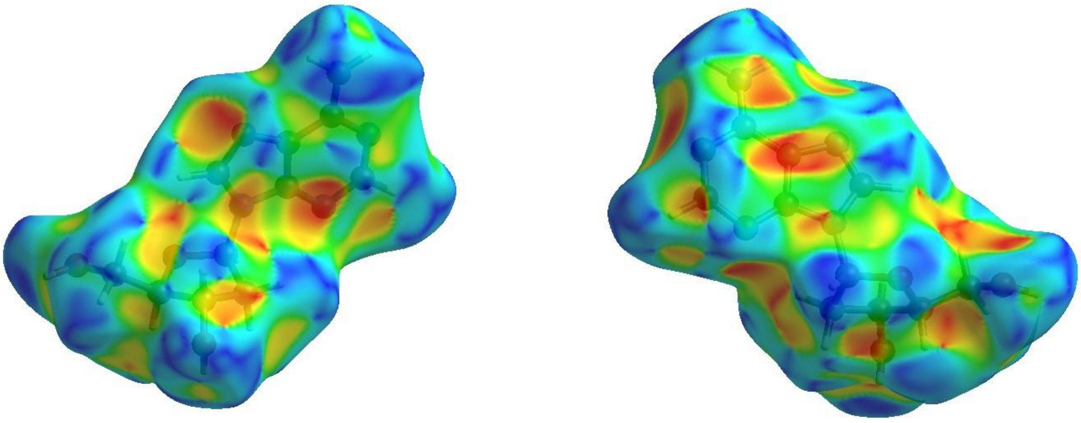

(*a*) (*b*)

Figure S9. Hirshfeld surface mapped over the shape index (-1.0 to 1.0 Å) of β-dA (**2**). (*a*) Front view. (*b*) Back view. Blue areas represent bumps, and red regions indicate hollows.

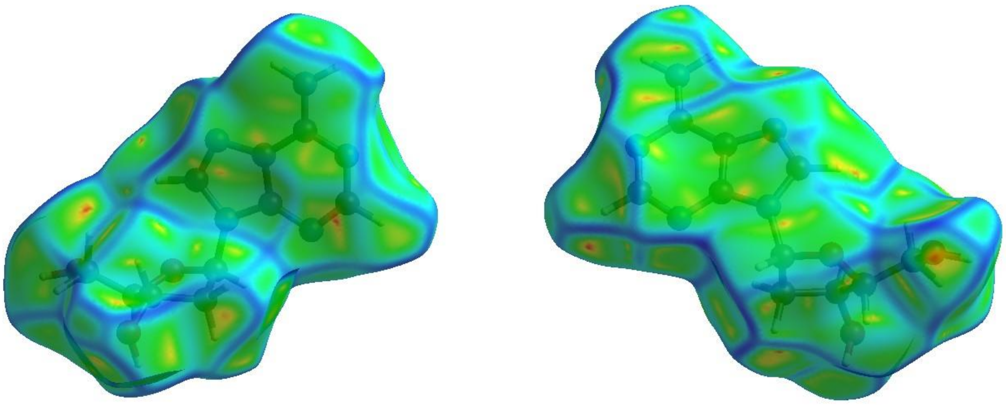

(*a*) (*b*)

Figure S10. Hirshfeld surface mapped over the curvedness (-4.0 to 4.0 Å) of β-dA (**2**). (*a*) Front view. (*b*) Back view.

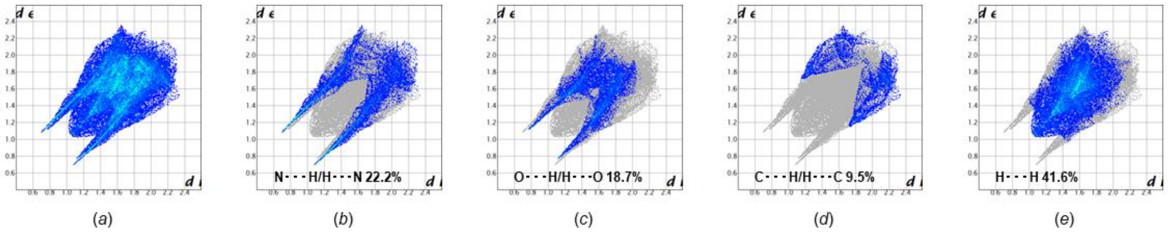

Figure S11. 2D fingerprint plots showing the percentage contributions of the various interactions to the total Hirshfeld surface area of β-dA (**2**). (*a*) All interactions. Resolved contacts of (*b*) N···H/H···N, (*c*) O···H/H···O, (*d*) C···H/H···C and (*e*) H···H.

Reference

Sato, T. (1984). *Acta Cryst.* C**40**, 880-882.
